# Design and engineering colloidal magnetic particles for nanoscale thermometry

**DOI:** 10.18416/IJMPI.2020.2009068

**Published:** 2020

**Authors:** A. J. Biacchi, T. Q. Bui, C. L. Dennis, S. I. Woods, A. R. Hight Walker

**Affiliations:** 1 Nanoscale Device Characterization Division, National Institute of Standards and Technology (NIST), Gaithersburg, MD, USA; 2 Sensor Science Division, National Institute of Standards and Technology (NIST), Gaithersburg, MD, USA; 3 Materials Science and Engineering Division, National Institute of Standards and Technology (NIST), Gaithersburg, MD, USA

## Abstract

Thermometry based on magnetic nanoparticles (MNPs) is an emerging technology that allows for remote temperature measurements throughout a volume that are impossible to achieve using conventional probe-based or optical methods. This metrology is based on the temperature-dependent nature of these particles’ magnetization; however, commercially available MNPs generally display insufficient magneto-thermosensitivity for practical use in applications near room temperature. Here we present engineered MNPs based on cobalt-doped ferrites developed for 200 K – 400 K thermometry applications. The synthesis relies on easily scalable solution chemistry routes, and is tunable to afford MNPs of controlled size and composition. These improved nanothermometers form the basis of our effort to develop a practical means for spatially resolved, 3D, high-sensitivity measurements of temperature based on AC magnetometry.

## I Introduction

**Figure 1:**
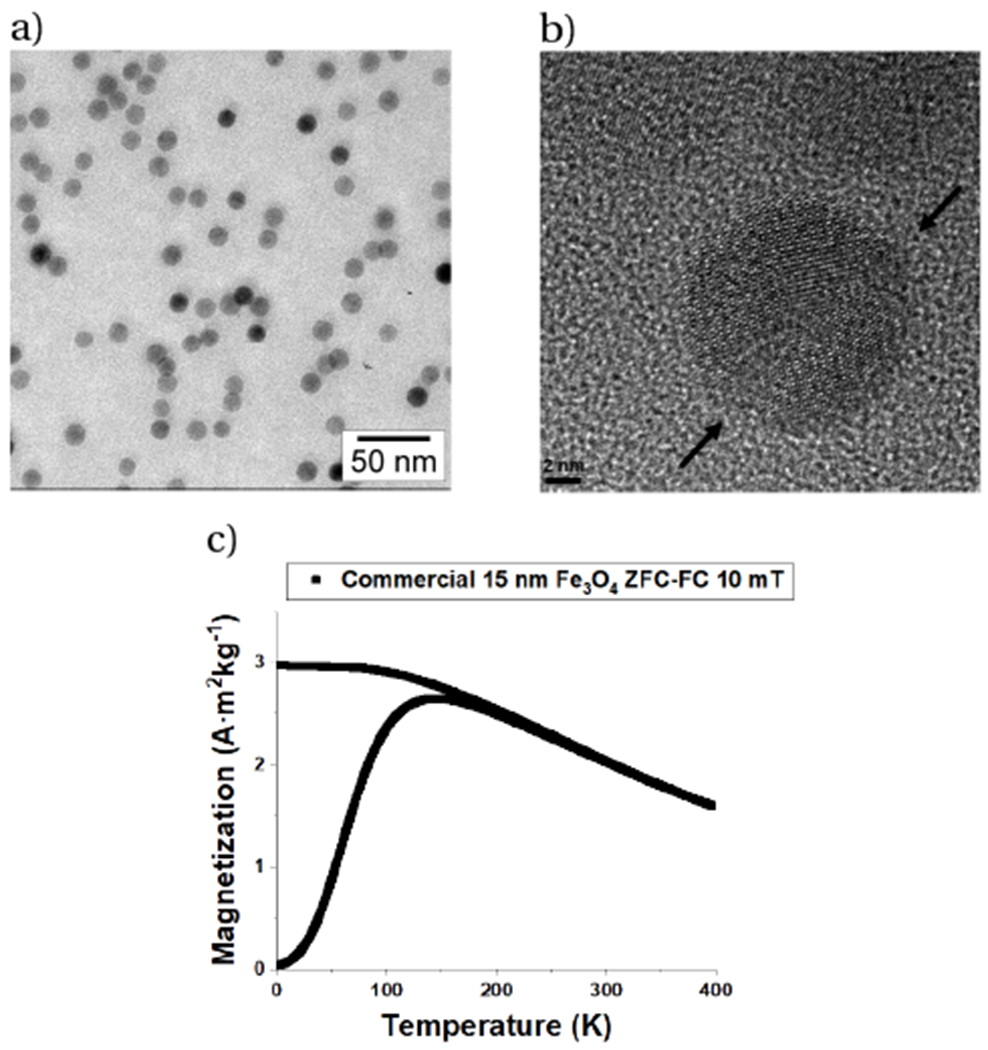
a) TEM micrograph of commercially available 15 nm Fe_3_O_4_ MNPs. Although highly monodisperse, b) high-resolution TEM analysis reveals multiple crystallographic domains within the particles. Consequently, these MNPs showed c) relatively modest DC magnetization values and strong thermosensitivity only below 150 K, which makes them undesirable for MNPT.

Accurate, local, remote, and real-time temperature measurements are essential for many technological applications. Conventional thermometry techniques are limited to remotely measuring the temperature of a microscale spot only on the surface; however, magnetic nanoparticle thermometry (MNPT) is currently being explored as a novel technique to measure temperature at comparable spatial resolution throughout a volume. Although progress has been made in the development of MNPT [1], the current technology lacks the sensitivity necessary for widespread application. Specifically, commercially available MNPs display only modest magnetization thermosensitivity, and primarily at temperatures too low for most applications (Fig. 1). Therefore, a prerequisite to practical volumetric nanothermometry is the development of MNPs with: a) improved magnetization temperature-dependence, b) high signal-to-noise ratio, and c) optimized performance close to room temperature.

Here we present solution-synthesized cobalt-doped ferrite nanoparticles and measure their magnetization dependence on the temperature of their local environment. Prudent control of the particles’ size and composition allows for different magnetic transition temperature regimes and sensitivities, including near room temperature. Particle monodispersity and crystalline domain size was also found to be controllable and yield stronger magnetization, thus providing a means to engineer these nanothermometers’ performance as a function of their structure.

Additionally, while most MNPT studies employ single-phase magnetic nanoparticles, we have begun engineering core-shell MNP heterostructures. Recent theory has predicted sharp temperature features in magnetization are induced in bilayer nanoparticles, where each layer is composed of a different magnetically coupled material [2]. We therefore target MNPs with a superparamagnetic core coupled to a nominally antiferromagnetic shell. Our preliminary studies of bimagnetic heterostructure nanoparticles suggest that these are a promising route towards MNPs with improved thermosensitivity.

## II Material and methods

We employed solution chemistries to synthesize a series of colloidal magnetic nanoparticles *via* the thermal decomposition of organometallic and/or halide precursors [3,4]. Briefly, metal acetoacetates or chlorides were dissolved in a benzyl ether solution matrix in the presence of a series of coordinating aliphatic surfactants such as oleic acid, oleylamine, and 1,2-hexadecanediol. This reaction mixture was degassed under vacuum and then heated to 550 K under a blanket of argon for two hours. The dark solution that developed indicated the formation of nanoparticles, which were subsequently collected by centrifugation and redispersed in hexanes.

Nanoparticles were thoroughly characterized using a variety of techniques. Structural and morphological properties were investigated using high-resolution transmission electron microscopy (HRTEM), X-ray diffraction (XRD), selected-area electron diffraction (SAED), small-angle X-ray scattering (SAXS), and Raman spectroscopy. Nanoparticle composition was elucidated using energy-dispersive X-ray spectroscopy (EDX), X-ray photoelectron spectroscopy (XPS), electron energy loss spectroscopy (EELS), and Mössbauer spectroscopy. The magnetic properties of the particles were determined using vibrating sample magnetometry (VSM), superconducting quantum interference device VSM (SQUID-VSM), and AC susceptibility. Their performance for MNPT was measured by conducting temperature-dependent differential measurements using a home-built AC magnetometer.

## III Results and discussion

**Figure 2:**
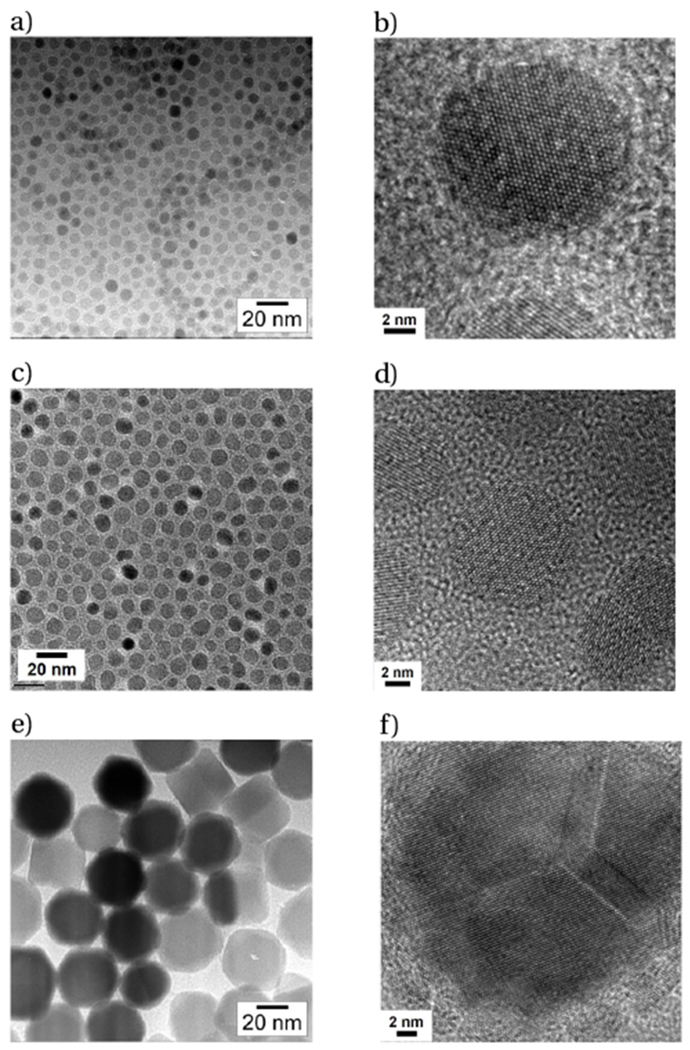
TEM micrographs of a) 7.5, c) 9.5, and e) 35 nm Co-doped ferrite MNPs. High-resolution TEM analysis indicated that b) the 7.5 nm particles and d) 9.5 nm particles are single-crystalline, while f) the 35 nm particles are polycrystalline.

**Figure 3:**
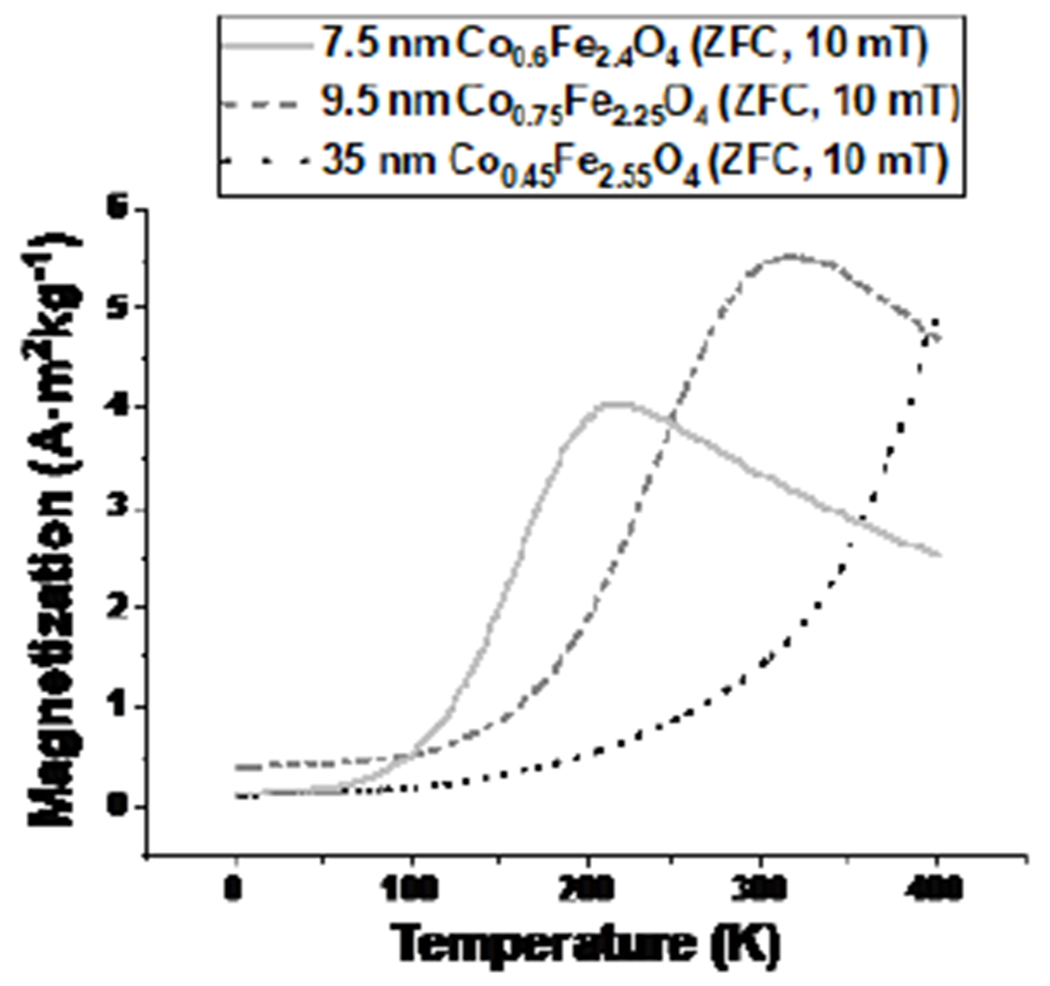
Zero field-cooled DC magnetization-temperature data collected at 10 mT for 7.5 nm, 9.5 nm, and 35 nm cobalt-doped ferrite showing improved and tunable thermosensitivity.

We synthesized cobalt-doped ferrite MNPs in numerous size regimes by carefully tailoring the concentrations and relative ratios of the starting reagents (Fig. 2a,c,e). Fine control of the temperature was found to be imperative to yielding highly monodisperse samples, with resulting size variances as low as 6.5 % by SAXS. Structural analysis revealed the MNPs to be phase-pure inverse spinel crystals. HRTEM confirmed that particles larger than ~20 nm in diameter formed twin boundaries and multiple domains, while smaller MNPs were single-crystalline (Fig. 2b,d,f). Chemical analysis of our particles confirmed cobalt doping of the ferrite, with combined Co^+2^/Co^+3^ incorporation levels ranging from 5 % to 25 %. Temperature-dependent DC magnetization measurements revealed two consistent trends: a) higher incorporation levels of Co resulted in regions of strong thermosensitivity occurring at higher temperatures and b) larger crystalline domain sizes gave rise to higher temperature regions of thermosensitivity (Fig. 3). We found that to produce cobalt-doped ferrite nanoparticles with strong magnetization thermosensitivity near room temperature required careful control of these sometimes-competing trends. Preliminary temperature-dependent AC magnetization studies confirm that these particles are a promising materials system for MNPT.

## IV Conclusions

Here we have shown that cobalt-doped ferrite MNPs are a promising material platform for room temperature thermometry due to their strong and tunable magnetic thermosensitivity in temperature ranges near ambient. Our preliminary temperature-dependent AC magnetization studies employing these MNPs reveal their ability to function as nanoscale probes for thermal measurement and control as well as self-consistent performance trends that can be correlated to their structural properties. Future efforts will focus on the fabrication of more complex bimagnetic heterostructured particles based on these cobalt-doped ferrites as part of our ongoing effort to engineer superior thermosensitivity into these MNPs.
